# Long-Term Efficacy and Cost-Effectiveness of Laser Tonsillotomy vs Tonsillectomy: A Secondary Analysis of a Randomized Clinical Trial

**DOI:** 10.1001/jamanetworkopen.2025.4858

**Published:** 2025-04-29

**Authors:** Justin Emile Raoul Edouard Wong Chung, Wilbert Bernhard van den Hout, Noud van Helmond, Peter Paul Germain van Benthem, Hendrikus Maria Blom

**Affiliations:** 1 Department of Otolaryngology Head and Neck Surgery, Hagaziekenhuis, The Hague, the Netherlands; 2 Department of Otolaryngology Head and Neck Surgery, Leiden University Medical Centre, Leiden, the Netherlands; 3 Department of Ophthalmology, University Medical Center Utrecht, Utrecht, the Netherlands; 4 Department of Ophthalmology, Maasziekenhuis Pantein, Boxmeer, the Netherlands; 5 Department of Biomedical Data Sciences, Leiden University Medical Centre, Leiden, the Netherlands; 6 Department of Anesthesiology, Cooper University Medical School of Rowan University, Cooper University Health Care, Camden, New Jersey; 7 Department of Otolaryngology Head and Neck Surgery, University Hospital Antwerp, Antwerp, Belgium

## Abstract

**Importance:**

The current treatment for adult tonsil disease, tonsillectomy (TE), may involve a burdensome recovery.

**Objective:**

To evaluate long-term efficacy (1- and 2-year efficacy) and cost-effectiveness of carbon dioxide (CO_2_) laser tonsillotomy (TO) vs TE.

**Design, Setting, and Participants:**

A prespecified secondary analysis of a randomized clinical trial was conducted in 5 Dutch hospitals. Participants included adults with persistent tonsil-related symptoms enrolled from January 25, 2018, to December 17, 2019. Data analysis was performed from January 5, 2025, to April 9, 2025.

**Interventions:**

Tonsillectomy under general anesthesia vs CO_2_ laser TO under local anesthesia.

**Main Outcomes and Measures:**

Intention-to-treat analysis on primary (persistent symptoms, defined as an answer of yes to the question of whether symptoms were still present, reported at 1 and 2 years) and secondary (symptom severity, patient satisfaction, quality-adjusted life-years [QALYs], and cost-effectiveness) outcomes.

**Results:**

In total, 98 patients were assigned to TO and 101 to TE; 98 were analyzed per group. The TO and TE groups were similar (69 [70%] vs 67 [68%] female; mean [SD] age, 29 [10] vs 30 [8] years). The most common symptom was sore throat with fever (34% vs 34%), with a baseline mean (SD) severity score of 57 (19) vs 59 (17) mm. At 1 year, 51.8% of patients assigned to TO had persistent symptoms vs 25.2% assigned to TE (odds ratio [OR], 3.2; 95% CI, 1.6-6.4; *P* < .001); at 2 years, 45.2% vs 19.7% had persistent symptoms (OR, 3.4; 95% CI, 1.7-6.7; *P* < .001). Symptom severity decreased significantly in both groups but was lower after TE at 1 year (14.8 vs 23.0 mm; mean difference, −8.1 mm; 95% CI, −14.8 to −1.5 mm; *P* = .02) and 2 years (10.8 vs 19.6 mm; mean difference, −8.8 mm; 95% CI, −14.7 to −2.9 mm; *P* = .001). Patient satisfaction was similar between groups; mean VAS scores were 79.0 (95% CI, 72.2-85.9) mm for TE and 69.3 (95% CI, 63.4-75.3) mm for TO at 1 year and 64.1 (95% CI, 55.7-72.5) mm and 64.4 (95% CI, 56.9-71.8) mm at 2 years. Similar proportions of participants would recommend the procedure at 1 year (79% TE vs 76% TO) and 2 years (71%, both). Both TE and TO demonstrated high cumulative QALYs at 2 years (EuroQol 5 Dimension: mean, 1.89 vs 1.84; mean difference, 0.05, *P* = .06; EuroQol Visual Analogue Scale: mean, 1.83 vs 1.81, mean difference, 0.02; *P* = .38). Tonsillotomy had lower overall costs ($869 vs $2363 for TE), with societal cost savings of $1925 (*P* = .001), and a 71% probability of cost-effectiveness at $25 907 per QALY (85%-93% in sensitivity analyses).

**Conclusions and Relevance:**

The findings of this trial suggest that both CO_2_ laser TO and TE under general anesthesia significantly reduced long-term symptoms, with greater reduction after TE. TO had lower cost and similar patient satisfaction. Based on these findings, CO_2_-laser TO appears to be a safe, effective, and cost-effective method for long-term relief of tonsil-related problems with excellent patient satisfaction.

**Trial Registration:**

trialregister.nl Identifier: NL 6866

## Key Points

**Question:**

How does carbon dioxide (CO_2_) laser tonsillotomy under local anesthesia (CO_2_ laser TO) compare with traditional tonsillectomy (TE) in terms of long-term efficacy and cost-effectiveness for adults with tonsil-related conditions?

**Findings:**

In a secondary analysis of a randomized clinical trial including 196 patients, TE was more effective in reducing long-term symptoms, with similar quality-adjusted life-years, while CO_2_ laser TO had lower costs. Both groups achieved significant symptom reduction and equal patient satisfaction, with CO_2_ laser TO being 71% to 93% likely to be cost-effective.

**Meaning:**

The results of this trial suggest that both TE and CO_2_ laser TO effectively reduce long-term tonsil-related symptoms; TE is more effective, but CO_2_ laser TO is a cost-effective option with shorter recovery and lower overall costs.

## Teaser

This secondary analysis of a randomized clinical trial compares the 1- and 2-year outcomes and cost-effectiveness of carbon dioxide laser tonsillotomy performed with local anesthesia with tonsillectomy performed under general anesthesia in adults in the Netherlands.

## Introduction

Tonsillectomy (TE) is a widely performed surgery under general anesthesia for adults with tonsil-related conditions such as recurrent tonsillitis, tonsillolithiasis, and airway obstruction, particularly when conservative treatment is ineffective. While TE is effective, it is invasive and associated with complications such as postoperative bleeding, infection, and substantial pain.^1,2^ Given its invasive nature, there is growing interest in less-invasive alternatives, such as carbon dioxide (CO_2_) laser tonsillotomy (TO), which can be performed under local anesthesia.^3^

Short-term studies suggest that CO_2_ laser TO offers safer, faster recovery and reduced postoperative pain compared with TE.^4,5^ The short-term results of the TOMTOM trial suggest that 77% of patients who underwent CO_2_ laser TO fully recovered within 2 weeks, compared with 57% of those who underwent TE, with a median time to return to work of 4.5 vs 12.0 days. Postoperative complications were also lower, with hemorrhage rates of 2% for CO_2_ laser TO compared with 12% for TE.^4^ Although tonsil-related symptoms persisted more frequently after CO_2_ laser TO (57% TO vs 35% TE), symptom severity was greatly reduced and patients report similarly high satisfaction in both study arms.^5^ Limited data on long-term outcomes and cost-effectiveness of CO_2_ laser TO leave uncertainty about the role of CO_2_ laser TO in clinical practice. This study compares the 1- and 2-year efficacy and cost-effectiveness of CO_2_ laser TO and TE under general anesthesia in adults in the TOMTOM study.

## Methods

### Study Design and Patients

This prespecified secondary analysis of original data examines a randomized clinical trial (TOMTOM study) that was conducted in 5 Dutch teaching hospitals. The present study adheres to the Consolidated Standards of Reporting Trials (CONSORT) reporting guideline. Results for the primary outcome of the TOMTOM trial have been previously reported.^5^ Approval by the local medical ethics committee (METC Zuid-West Holland) was obtained. Patients were recruited from January 25, 2018, to December 17, 2019. All patients provided written informed consent; no financial compensation was provided. Adults with chronic or recurrent tonsillitis, halitosis, tonsillolithiasis, dysphagia, and sleep apnea were included if their symptoms were unresponsive to conservative treatments. Key exclusion criteria included Friedman grade 4 tonsil size, contraindications to anesthesia, and pregnancy. Full patient inclusion and exclusion details are provided in the trial protocol (Supplement 1) and in eMethods in Supplement 2.

### Randomization

Patients were randomized to either CO_2_ laser TO or TE using computer-generated stratification based on primary tonsil concern. Patients could undergo additional surgical treatments if clinically necessary for a pragmatic and ethical trial design. Data collection continued even if patients opted out of their assigned treatment.

### Procedures

The CO_2_ laser TO procedure was performed under local anesthesia with xylocaine and adrenaline, following standard safety protocols. A step-by-step video protocol for this intervention has been previously published.^6^ Classic TE was performed under general anesthesia using standard dissection and electrosurgical techniques. Procedure protocols and postoperative pain medication details can be found in Supplement 1.

### Data Collection

Outcomes were collected via digital questionnaires at 1 and 2 years post surgery, measuring tonsil-related symptoms, quality of life (EuroQol 5 Dimension [EQ-5D], range 1 [representing full health] to 0 [representing death]; EuroQol Visual Analogue Scale [EQ-VAS], vertical visual analogue scale with values between 1 [best imaginable health] and 0 [worst imaginable health]),^7^ health care use, Work Productivity and Activity Impairment,^8^ and patient satisfaction with treatment (visual analog scale [VAS], range 0-100 mm, with higher scores indicating greater satisfaction). Recovery times were collected at 2 and 6 weeks. Missing data were handled using multiple imputation. More details can be found in eMethods in Supplement 2.

### Economic Evaluation

A cost-utility analysis was conducted from a societal perspective, at 2023 price levels, with a 2-year horizon. Utility reflects the value of quality of life (scale 0-1) and was calculated using the Dutch tariff for the EQ-5D^9^ and EQ-VAS data.^10^ A cost-price analysis was performed for both procedures. All costs were analyzed in euros and subsequently converted to US dollars using the 2024 Organisation for Economic Co-operation and Development Purchasing Power Parity for gross domestic product (€0.772=$1). Quality-adjusted life years (QALYs) were derived from the area under the utility curves over the follow-up period. EQ5-VAS scores were analyzed as 0-1 scores. Other tonsil-related health care, absenteeism, and presenteeism at work were patient-reported. Three sensitivity analyses were performed in which costs were limited to health care costs (instead of societal costs), patients without registered TE or CO_2_ laser TO were assumed to have had TE (instead of assuming no procedure), and QALYs were calculated from the EQ-VAS (instead of the EQ-5D index score). Full economic evaluation details are available in eMethods in Supplement 2.

### Statistical Analysis

Data analysis was conducted from January 5, 2025, to April 9, 2025. The target sample size was determined for previously published short-term outcomes of this study.^5^ Based on prior short-term outcomes,^4^ the target sample size was 190 patients (95 per group) to achieve 80.2% power at a .05 significance level. This allowed for the detection of a median recovery time of 8.0 days for CO_2_ laser TO, compared with 13.5 days for TE, with a 14-day observation period.

Baseline characteristics were summarized as means (SDs) or counts (percentages). Long-term outcomes were analyzed on an intention-to-treat basis, using unpaired *t* tests for pooled means and logistic regression for binary outcomes. Changes from baseline were assessed with paired *t* tests. All tests were 2-sided, with a significance level of *P* < .05. Analyses were conducted using SPSS, version 27 (IBM Corp), with annual external data monitoring ensuring data quality. Additional statistical methods are presented in the protocol in Supplement 1.

## Results

### Patient Inclusion and Disposition

**Figure 1.**
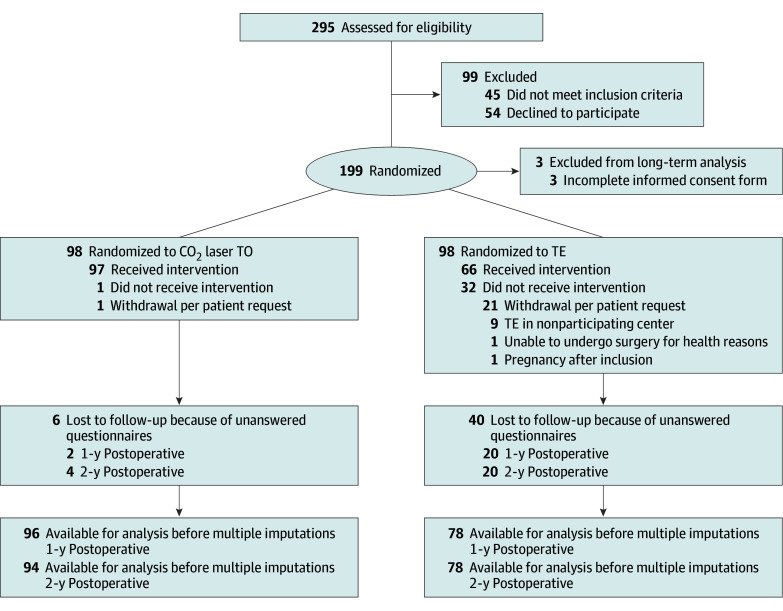
Trial Flow Diagram CO_2_ indicates carbon dioxide; TE tonsillectomy; and TO, tonsillotomy.

Of the 199 patients randomized, 98 were assigned to CO_2_ laser TO and 101 to TE. After excluding 3 patients in the TE group due to informed consent discrepancies, 196 patients were included in the final analysis (Figure 1). The CO_2_ laser TO and TE groups were similar (TO: 69 [70%] female, 29 [30%] male vs TE: 67 [68%] female, 31 [32%] male; mean [SD] age, 29 [10] vs 30 [8] years).

Baseline characteristics were comparable between the TO and TE groups in terms of chief tonsil symptoms, with sore throat with (34% vs 34%) and without fever (32% vs 32%) being the most common, followed by tonsillolithiasis (33% vs 32%). The self-reported severity of tonsil symptoms (mean [SD] severity score) was similar between groups (mean [SD], 57 [19] mm for TO vs 59 [17] mm for TE), and most patients rated their symptoms as moderate or severe. Smoking status also showed a similar distribution between groups, with approximately 18% to 14% current smokers, 25% to 16% former smokers, and 58% to 47% never smokers for CO_2_ laser TO vs TE (eTable 1 in Supplement 2). A total of 163 patients (82%) received their assigned treatment.

In the CO_2_ laser TO group, 17 patients required a second treatment for residual symptoms, 9 switched to TE for recurrent symptoms. In the TE group, 32 did not undergo the assigned procedure, and 9 patients reported to have received TE at nonparticipating centers. The primary reason for withdrawal in the TE group was patients opting out after randomization (Figure 1). More censored patients were noted in the TE vs TO group for full recovery (35 vs 22), return to work (8 vs 5), and analgesic use (16 vs 3).

### Efficacy

**Figure 2.**
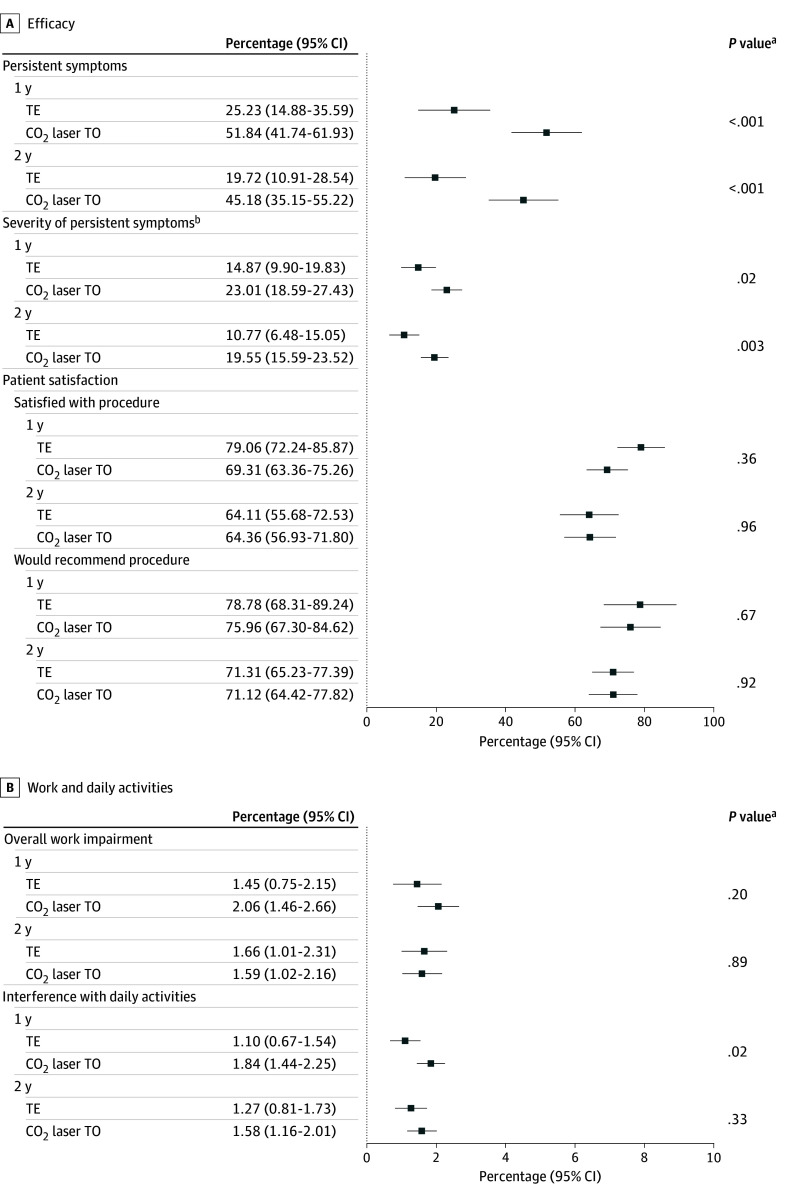
Long-Term Outcome Measures One- and 2-year measures of efficacy (A) and work and daily activities (B). Work impairment was assessed only in employed patients. TE, tonsillectomy; TO, tonsillotomy. ^a^*P* values are based on independent *t* tests for continuous variables and logistic regression for binary outcome variables. ^b^Measured on a vertical visual analogue scale with values between 100 (best imaginable health) and 0 (worst imaginable health).

One year after surgery, 25.2% of patients who underwent TE reported persistent symptoms (reported as yes or no by patients on postoperative evaluation) compared with 51.8% of those in the CO_2_ laser TO group (odds ratio [OR], 3.2; 95% CI, 1.6-6.4; *P* < .001) (Figure 2). At 2 years, 19.7% of patients in the TE group vs 45.2% of those in the TO group reported persistent symptoms (OR, 3.4; 95% CI, 1.7-6.7; *P* < .001).

Symptom severity in patients with remaining symptoms decreased significantly in both groups, but remained lower after TE at both 1 year (mean VAS score, 14.8; 95% CI, 9.9-19.8 vs mean, 23.0; 95% CI, 18.6-27.4 mm; mean difference, −8.1; 95% CI, −14.8 to −1.5 mm; *P* = .02) and 2 years (mean VAS score, 10.8; 95% CI, 6.5-15.1 vs mean, 19.6; 95% CI, 15.6-23.5 mm; mean difference, −8.8; 95% CI, −14.7 to −2.9 mm; *P* = .001). Symptom severity significantly decreased from baseline after 1 year in both the CO_2_ laser TO group (mean baseline, 56.6 vs 1 year, 23.0 mm; mean difference, 33.6; 95% CI, 28.5-38.7 mm; *P* < .001) and the TE group (mean baseline, 59.2 vs 1 year, 14.9 mm; mean difference, 44.3; 95% CI, 38.1-50.5 mm; *P* < .001). Among patients with persistent symptoms at 1 year, self-reported severity shifted toward mild and moderate after CO_2_ laser TO (mild 26.4%, moderate 22.0%, severe 3.5%) and TE (mild 18.0%, moderate 4.7%, severe 2.5%).

At 2 years, symptom severity continued to decrease in both groups: CO_2_ laser TO (mean baseline, 56.6 vs 2 years, 19.6 mm; mean difference, 37.1; 95% CI, 31.2-43.0 mm; *P* < .001) and TE (mean baseline, 59.2 vs 2 years, 10.8 mm, mean difference, 48.4; 95% CI, 42.8-54.0 mm; *P* < .001). Patients with persistent symptoms after 2 years experienced mostly mild and moderate symptoms in both the CO_2_ laser TO (mild 28.4%, moderate 14.7%, severe 2.1%) and TE (mild 9.0%, moderate 9.4%, severe 1.4%) groups.

### Patient Satisfaction

There was no significant difference 1 year after surgery in patient satisfaction (mean score, 79.0; 95% CI, 72.2-85.9 mm for TE and mean, 69.3; 95% CI, 63.4-75.3 mm for TO; *P* = .36) and 2 years post surgery (mean VAS score, 64.1; 95% CI, 55.7-72.5 mm for TE and mean, 64.4; 95% CI, 56.9-71.8 mm for TO; *P* = .96). Almost equal percentages of patients would recommend their surgery to others at both 1 year (TE, 79% vs TO, 76%; OR, 0.8; 95% CI, 0.4-1.9; *P* = .67) and 2 years (both 71%; OR, 1.0; 95% CI, 0.5-2.1; *P* = .92).

### Work and Daily Activities

Work impairment was minimal in both the CO_2_ laser TO and TE groups at 1 year (TO: mean, 2.1%; 95% CI, 1.5%-2.7%; vs TE: mean, 1.5%; 95% CI, 0.8%-2.2%; mean difference, −0.6%; 95% CI, −1.5%-0.5%; *P* = .20) and 2 years (TO: mean, 1.6%; 95% CI, 1.0%-2.2% vs TE: 1.7%; 95% CI, 1.0%-2.3%; mean difference, 0.1%; 95% CI, −0.8% to 0.9%; *P* = .93). Absolute interference with daily activities was similarly low in both groups at long-term follow-up, but was statistically lower in the TE group at 1 year (TE: mean, 1.1%; 95% CI, 0.7%-1.5% vs TO: mean, 1.8%; 95% CI, 1.4%-2.2%; mean difference, −0.7%; 95% CI, −1.3% to −0.1%; *P* = .02) but not 2 years postoperatively (mean, 1.3; 95% CI, 0.8-1.7 mm after TE vs 1.6; 95% CI, 1.2-2.0 mm after TO; mean difference, −0.3; 95% CI, −0.9 to 0.3 mm; *P* = .33).

### Utilities and QALYs

All postbaseline health-related quality of life utility measures showed small differences between the TE and CO_2_ laser TO groups (Table 1; eFigure in Supplement 2), mostly without statistical significance. Over 2 years, the cumulative QALY difference was 0.05 according to the EQ-5D (TO vs TE: means, 1.89 vs 1.84; *P* = .06; mean difference, 0.05; 95% CI, −0.00 to 0.11) and 0.02 according to the cumulative EQ-VAS (TO vs TE: means, 1.83 vs 1.81; *P* = .38; mean difference, 0.02; 95% CI, −0.03 to 0.07).

**Table 1.** Utilities and QALYs, Over Time and by Randomization Group

Characteristic	Utility measures		Difference (95% CI)	*P* value
	Tonsillotomy (n = 98)	Tonsillectomy (n = 98)		
**EQ-5D utilities**				
Baseline	0.87	0.90	0.03 (−0.01 to 0.07)	.15
6 wk	0.93	0.95	0.02 (−0.01 to 0.05)	.17
6 mo	0.92	0.94	0.02 (−0.02 to 0.06)	.36
12 mo	0.91	0.94	0.03 (−0.01 to 0.07)	.21
24 mo	0.93	0.97	0.04 (−0.00 to 0.07)	.03
**EQ-5D QALYs**				
Year 1	0.92	0.94	0.02 (−0.01 to 0.05)	.14
Year 2	0.92	0.95	0.03 (0.00 to 0.06)	.05
Both	1.84	1.89	0.05 (−0.00 to 0.11)	.06
**EQ-VAS utilities**				
Baseline	0.90	0.87	−0.02 (−0.06 to 0.01)	.21
6 wk	0.91	0.91	0.00 (−0.04 to 0.04)	.94
6 mo	0.89	0.92	0.03 (−0.01 to 0.07)	.20
12 mo	0.91	0.92	0.01 (−0.02 to 0.04)	.65
24 mo	0.91	0.92	0.01 (−0.01 to 0.04)	.36
**EQ-VAS QALYs**				
Year 1	0.90	0.91	0.01 (−0.02 to 0.04)	.40
Year 2	0.91	0.92	0.01 (−0.01 to 0.03)	.44
Both	1.81	1.83	0.02 (−0.03 to 0.07)	.38

Abbreviations: EQ-5D, EuroQol 5 Dimension; EQ-VAS, EuroQol Visual Analogue Scale; QALYs, quality-adjusted life-years.

### Costs

Costs per CO_2_ laser TO procedure were estimated at less than half the costs of the TE procedure ($869 vs $2363) (eTable 2 in Supplement 2). The difference in average surgery costs per patient was estimated at $304 (95% CI, $74-$534) (Table 2). This relatively small difference was due both to untreated patients in the TE group and repeated treatment in the CO_2_ laser TO group.

**Table 2.** Average 2-Year Tonsil-Related Health Care and Societal Costs Per Patient, by Randomization Group

Variable	Tonsillotomy (n = 98)			Tonsillectomy (n = 98)			Differences	
	%	Volume	Costs (SD), $	%	Volume	Costs (SD), $	Costs, $	*P* value
Tonsillectomy	9	0.09	185 (580)	76	0.76	1519 (865)	1334	<.001
CO_2_-laser tonsillotomy	98	1.19	1030 (472)	0	0.00	0 (0)	−1030	<.001
Total surgery costs	99	1.29	1215 (754)	76	0.76	1519 (865)	304	.01
Pain medication	56	NA	10 (28)	34	NA	9 (31)	−1	.73
Antibiotics	25	NA	9 (25)	6	NA	1 (9)	−8	.01
General practitioner	37	0.94	52 (95)	32	0.71	39 (79)	−13	.38
Speech therapist	5	0.20	10 (47)	2	0.19	9 (82)	0	.96
Alternative care	3	0.09	9 (60)	1	0.02	3 (22)	−6	.27
Company physician	4	0.10	13 (75)	0	0.01	1 (26)	−12	.17
Emergency department	4	0.06	27 (136)	6	0.06	27 (109)	0	.99
Hospitalization^a^	3	0.21	168 (1315)	7	0.13	102 (573)	−65	.67
Total nonsurgery health care	NA	NA	299 (1567)	NA	NA	192 (624)	−106	.55
Total health care costs	NA	NA	1514 (1760)	NA	NA	1711 (1053)	197	.35
Absenteeism from work^b^	73	2.2	810 (1083)	72	5.8	2154 (3163)	1345	.003
Presenteeism at work^b^	56	0.7	254 (636)	47	1.7	637 (1334)	383	.03
Total productivity	76	2.9	1063 (1328)	73	7.5	2791 (3574)	1728	.001
Total societal costs (SD)	NA	NA	2578 (2223)	NA	NA	4503 (3777)	1925	.001

Abbreviation: NA, not applicable.

^a^ Volume is in hospital days.

^b^ During 6 weeks after the initial procedure. Volume is in working day equivalents.

Other health care costs were consistently higher in the CO_2_ laser TO group, but these differences were not statistically significant and were limited in size. The difference in total health care costs was estimated at a nonsignificant and small amount of $197 (95% CI, −$223 to $618).

Both absence from work and reduced productivity while at work were significantly higher in the TE group during 6 weeks after the initial procedure, with an estimated combined cost difference of $1728 (95% CI, $766-$2690). As a result, the total societal costs were also significantly higher in the TE group, by $1925 (95% CI, $854-$2997).

### Cost-Effectiveness

**Figure 3.**
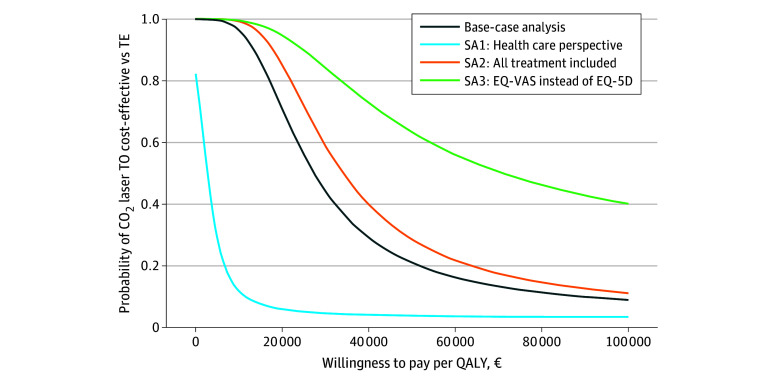
Cost-Effectiveness Acceptability Curves Cost-effectiveness acceptability curves show the probability that carbon dioxide (CO_2_) laser tonsillotomy (TO) is cost-effective compared with tonsillectomy (TE), depending on the willingness to pay per quality-adjusted life-year (QALY). Different curves show the base-case analysis and 3 sensitivity analyses. To convert euros to US dollars, the 2024 Organisation for Economic Co-operation and Development Purchasing Power Parity for gross domestic product (€0.772=$1) applies. SA1: only health care costs; SA2: assuming unregistered cases received TE; and SA3: EuroQol 5 Dimension (EQ-5D) and EuroQol Visual Analogue Scale (EQ-VAS).

Figure 3 shows the probability that CO_2_ laser TO is cost-effective compared with TE, depending on the willingness to pay per QALY. For this relatively mild condition, the appropriate cost-effectiveness threshold in the Netherlands is $25 907 per QALY.^11^ At that threshold, CO laser TO is 71% likely to be cost-effective compared with TE. The estimated cost-utility ratio is $36 269 per QALY (95% CI, $11 658-infinity), favoring the less-expensive CO_2_ laser TO.

Three sensitivity analyses were conducted to account for potential biases. In the first sensitivity analysis, only health care costs were considered, excluding productivity costs (SA1 in Figure 3). At a threshold of $25 907 per QALY, this reduced the likelihood of CO_2_ laser TO being cost-effective to 6%, highlighting the importance of productivity costs in cost-effectiveness.

In the TE group, 24% of patients had no registered TE, likely due to dropouts after not being assigned the CO_2_ laser TO. These patients may have received TE at a more convenient hospital. In the second sensitivity analysis, all unregistered cases were assumed to have received TE, which increased the surgery cost difference by $472, resulting in a total difference of $776 (95% CI, $627-$926). This raised the likelihood of CO_2_ laser TO being cost-effective from 71% to 85% at a $25 907 per QALY threshold (SA2 in Figure 3).

In the third sensitivity analysis (SA3 in Figure 3), QALYs were calculated using the EQ-VAS instead of the EQ-5D, reducing the QALY advantage for TE. This increased the probability of CO_2_ laser TO being cost-effective to 93% at a $25 907 per QALY threshold, with an estimated cost-utility ratio of $91 969 per QALY (95% CI, $24 611-infinity), favoring the less-expensive CO_2_ laser TO.

## Discussion

To our knowledge, this secondary analysis of a randomized clinical trial is the largest to compare long-term outcomes of CO_2_ laser TO and TE in adults. Previous findings reported that CO_2_ laser TO led to faster, less-painful recovery and lower postoperative hemorrhage compared with TE. Although symptom persistence was higher with CO_2_ laser TO at 6 months, both groups experienced reduced symptom severity, improved quality of life, and high patient satisfaction.^5^ At 1- and 2-year follow-ups, patients who underwent TE reported fewer and milder symptoms than those who received CO_2_ laser TO. Both groups with residual symptoms experienced significant symptom reduction to clinically nonrelevant levels after 2 years (VAS <20 mm). Satisfaction, willingness to recommend surgery, and work productivity impact were similar across both time points. While CO_2_ laser TO had slightly lower QALYs, it significantly reduced surgery and productivity costs, with a 71% to 93% likelihood of being cost-effective. These findings are consistent with the 6-month data, where 57% of patients in the CO_2_ laser TO group and 35% of those in the TE group had persistent symptoms, with 13% of patients in the CO_2_ laser TO group needing a second treatment.^5^ Both groups showed reduced symptom severity at 6 months, which continued through the 1- and 2-year follow-ups. Quality of life improvements also persisted. Although patients in the TE group had slightly higher satisfaction at 6 months, this difference diminished over time. A similar percentage of patients in both groups would recommend their surgeries. These results address gaps in the literature, which emphasize short-term benefits of TO but with limited evidence on long-term outcomes.^3,12^ The higher occurrence of residual symptoms after CO_2_ laser TO is likely due to incomplete tonsil removal, unlike TE. Retaining the tonsillar bed with major nerves and blood vessels allows CO_2_ laser TO to be performed under local anesthesia and reduces postoperative bleeding, which lowers the need for surgical revision due to hemorrhage and reduces postoperative pain and recovery time.^5^ However, the management of postoperative bleeding may vary across institutions, particularly in the threshold for performing surgical revisions. This highlights the importance of considering institutional practices and patient preferences when counseling on TO vs TE. Some patients required a second procedure within 6 months, resulting in significant and lasting symptom improvement, underscoring the importance of adequate tissue removal for successful CO_2_ laser TO.

To date, few studies have compared the long-term efficacy of TE and TO in adults. A review reported no significant difference in outcomes over up to 6 years in 5 of 6 studies, although variations in surgical methods, indications, and criteria complicated comparisons, and some studies lacked quality.^3^ A previous nonrandomized cohort study reported 72.5% of patients were symptom-free 1 year after CO_2_ laser TO vs 97.2% after TE, with similar satisfaction.^4^ Outside the adult context, longer-term follow-up in children support the durability of TO. A 12-year follow-up study in children found no significant differences between TO and TE in disease-specific quality of life, throat infections, or satisfaction rates, with most patients free from tonsil-related issues.^13^ Similarly, a 6-year randomized study in children comparing CO_2_ laser TO with TE found equally stable outcomes in snoring, apneas, and infections, with no significant differences between groups. Patient satisfaction and health improvements were high in both study arms.^14^

The cost-effectiveness of TO in adults has been minimally studied, with some research suggesting it may be more cost-effective than TE.^15,16^ However, to our knowledge, this study provides the only systematic evaluation of TO cost-effectiveness in adults to date. In contrast, TE vs conservative management for recurrent tonsillitis in adults has been extensively studied, with a large randomized clinical trial showing TE to be both clinically effective and cost-effective compared with conservative management.^2^ While our study lacked a conservative management arm, it is plausible that immediate CO_2_ laser TO is also cost-effective compared with conservative management. In this study, the costs for CO_2_ laser TO were considerably lower than for TE ($869 vs $2363). However, due to additional surgeries in the TO group, the total cost difference was reduced to $304. This likely underestimates the true cost difference, as some patients in the TE group may have received TE elsewhere during the study period. Given that over 100 000 tonsillectomy procedures are performed annually in the US alone, the potential cost savings demonstrated by CO_2_ laser TO could have substantial societal and health care system implications.^17^ Beyond cost savings from avoiding general anesthesia, CO_2_ laser TO frees operating rooms for procedures requiring anesthesia. This logistical advantage is useful, especially with the growing global backlog of surgeries.^18^ To our knowledge, this is the largest randomized clinical trial and the first to evaluate the cost-effectiveness of CO_2_ laser TO in adults, showing a 71% likelihood of being cost-effective compared with TE at a $25 907 per QALY threshold. Sensitivity analyses highlight the importance of productivity costs, as focusing solely on health care costs reduces this likelihood to 6%, while accounting for patients with unregistered TE increases it to 85%. These results rely on the EQ-5D tool for health-related quality of life measurement, which may not fully capture tonsil-related issues. The EQ-VAS, reflecting patients’ overall health perceptions, could provide a more comprehensive assessment.^19,20^ Using the EQ-VAS to calculate QALYs raises the likelihood of CO_2_ laser TO being cost-effective to 93%.

While there are many different methods used for TO surgeries, we chose to use a CO_2_ laser. The CO_2_ laser efficiently cuts and evaporates tissue with photothermal hemostasis, minimizing surrounding tissue damage, edema, and scarring compared with other methods.^21,22^

### Limitations

This study has limitations. The TE group had a higher withdrawal rate, but since withdrawals were not based on treatment outcomes, bias is unlikely. Baseline characteristics of treated (both within and outside the study) and withdrawn patients showed no significant differences, suggesting minimal withdrawal bias. The higher TE withdrawal rate may reflect reluctance toward more invasive surgery, and the intention-to-treat analysis mirrors clinical practice patient burden and treatment effect. Sensitivity analysis assuming all withdrawals received TE elsewhere increased the surgical cost difference. Patients were asked about additional treatments during follow-up, but not all who opted out of TE completed questionnaires, potentially missing some TE treatments conducted outside the study. Multiple imputations addressed potential missing data bias. Further limitations are that nonsurgical health care and productivity were patient-reported and could be subject to bias, as patients were aware of their treatment allocation. The study setting may not reflect other health care systems with different cost-effectiveness thresholds than the $25 907 per QALY used. Dutch postprocedure management practices and costs may not be entirely generalizable internationally due to differences in health care systems and guidelines, although the core findings, such as quicker recovery, reduced need for general anesthesia, and cost-effectiveness, are likely applicable in similar settings. Additionally, we did not specifically analyze the potential impact of procedural timing on absenteeism. While this factor could influence the results, any effect is likely minimal.

## Conclusions

Based on results of this randomized clinical trial, CO_2_ laser TO appears to be ideal for adult patients prioritizing quicker recovery and less postoperative discomfort. It suits those unable or unwilling to undergo general anesthesia, need minimal disruption to daily activities, or are apprehensive about the invasiveness of TE.

In addition, CO_2_ laser TO is recommended for patients with mild to moderate recurrent tonsil-related symptoms, where full tonsil removal may not always be necessary. Although some residual tissue and symptoms may remain, TO significantly reduces symptom severity to clinically nonrelevant levels, with low postoperative risk and low health care cost. Its reduced need for in-hospital care and preservation of tonsillar structure might align better with health care goals of individual patients.

For patients who wish to avoid the possibility of a secondary procedure, traditional TE may be the more appropriate choice. Careful patient selection and counseling about the potential for residual symptoms and a secondary procedure are essential to optimizing outcomes and satisfaction. This personalized approach, backed by the major economic benefits demonstrated in this study, underscores the value of integrating CO_2_ laser TO into treatment strategies for persistent tonsil-related afflictions in adults.

This study’s long-term follow-up showed that CO_2_ laser TO was less effective than TE in fully resolving tonsil issues but led to a substantial decrease in symptoms for all patients with residual symptoms, resulting in similar patient satisfaction. A slight advantage in 2-year QALYs was noted with TE, but CO_2_ laser TO was less costly, with lower societal costs due to reduced work absence and productivity loss. Based on these findings, CO_2_ laser TO appears to be a safe, effective, and cost-effective method for long-term relief of tonsil-related problems with excellent patient satisfaction.

## Notes

Supplement 1.
Trial Protocol

Supplement 2. **eTable 1.** Baseline Demographic and Clinical Characteristics in Tonsillotomy and Tonsillectomy Groups **eTable 2.** Cost Price of the Tonsillectomy and CO_2_-Laser Tonsillotomy Procedures (in €) **eFigure.** Utilities Over Time, by Randomization Group

Supplement 3.
Data Sharing Statement
