# Supplementary material for: Long-Term Efficacy and Cost-Effectiveness of Laser Tonsillotomy vs Tonsillectomy: A Secondary Analysis of a Randomized Clinical Trial
Source: JAMA Netw Open. 2025 Apr 29;8(4):e254858. doi: 10.1001/jamanetworkopen.2025.4858 (PMC12042055; doi:10.1001/jamanetworkopen.2025.4858)
Supplement: Supplement 2. — eTable 1. Baseline Demographic and Clinical Characteristics in Tonsillotomy and Tonsillectomy Groups eTable 2. Cost Price of the Tonsillectomy and CO2-Laser Tonsillotomy Procedures (in €) eFigure. Utilities Over Time, by Randomization Group [file jamanetwopen-e254858-s002.pdf]

Supplemental Online Content

Wong Chung JERE, van den Hout WB, van Helmond N, van Benthem PPG, Blom HM. Long-term efficacy and cost-effectiveness of laser tonsillotomy vs tonsillectomy: a secondary analysis of a randomized clinical trial. *JAMA Netw Open.* 2025;8(4):e254858. doi:10.1001/jamanetworkopen.2025.4858

eMethods

eReferences

eTable 1. Baseline Demographic and Clinical Characteristics in Tonsillotomy and Tonsillectomy Groups

eTable 2. Cost Price of the Tonsillectomy and CO_2-Laser Tonsillotomy Procedures (in €)

eFigure. Utilities Over Time, by Randomization Group

This supplemental material has been provided by the authors to give readers additional information about their work.

© 2025 Wong Chung JERE et al. *JAMA Network Open*.

eMethods in detail

Study design and patients

The TOMTOM study (Netherlands Trial Register, Identifier: NL6866 [NTR7044]), conducted in 5 Dutch teaching hospitals, adhered to CONSORT guidelines and received approval from The Hague's Research Ethics Committee (METC Zuid-West Holland, NL57496.098.16). Short-term follow-up results of this study were previously published.[1] Patients were recruited from January 2018 to December 2019. All patients provided written informed consent.

The study included adult patients with chronic or recurrent tonsillitis, halitosis, tonsillolithiasis, dysphagia, and sleep apnea attributed to tonsillar problems. Tonsil symptoms had to be inadequately responsive to conservative treatment methods, necessitating surgical intervention as per the prevailing treatment guidelines in the Netherlands.[2] Exclusion criteria comprised inability to complete all trial procedures and follow-up visits, inability to keep the mouth open continuously for at least 5 seconds or relax the jaw for 30 minutes, and inadequate exposure of the entire tonsil on physical examination, including Friedman grade 4 (kissing) tonsils. With kissing tonsils, the laser must be directed straight toward the back of the throat during the initial phase of the laser treatment, increasing the risk of damaging the posterior pharyngeal wall due to the lack of a protective buffer of underlying tonsil tissue. Additional exclusion criteria included: history of peritonsillar abscess, coagulation disorders (including anticoagulant use), contraindications for local or general anesthesia, evident tonsil asymmetry or signs suggesting potential (pre-)malignant oropharyngeal neoplasms, immunodeficiency, and pregnancy.

Randomization

Computer-generated random numbers were used for assigning patients randomly to either CO_2-laser TO or TE, with stratified randomization based on their primary tonsil concern. Patients were allowed to undergo additional surgical treatments if clinically necessary to maintain a pragmatic and ethical

© 2025 Wong Chung JERE et al. *JAMA Network Open*.

randomized clinical trial design, and those who opted out of their assigned treatment were requested to allow continued data collection on tonsil symptoms and subsequent surgeries.

Procedures

CO2 Laser Tonsillotomy under Local Anesthesia

CO_2-laser TO was performed in ambulatory intervention rooms, adhering to standard laser safety guidelines.[3] Prior to surgery, each patient received oral acetaminophen (1 gram). Local anesthesia of the tonsil was achieved with xylocaine (2%) containing adrenaline (1:80,000 units), up to a maximum of 5.4 mL. For patients with a significant residual gag reflex, xylocaine (10%) was sprayed on the peritonsillar area. Patients were instructed to breathe deeply; during exhalation, with the tongue depressed, the tonsil crypts were evaporated in a sweeping motion until complete cryptolysis was achieved. In case of bleeding, coagulation was performed by adjusting the laser out of focus. A step-by-step video protocol for this intervention has been previously published.[4] All CO_2-laser TO procedures were conducted at the primary clinical study center, with participating centers located within a two-hour driving distance, facilitating patient access to treatment.

Patients assigned to CO_2-laser TO were instructed to gradually diminish their gag reflex by brushing their tongue base and tonsils with a toothbrush during regular teeth brushing two weeks before surgery.

Classic Dissection Tonsillectomy

Classic dissection tonsillectomy procedures were conducted at all study centers. Patients were placed in a supine position and administered general anesthesia with endotracheal intubation. A McIvor retractor was then applied, and the superior pole of the tonsil was grasped using an Allis clamp. To expose the tonsil an incision on the anterior pillar of the tonsil was made and the tonsil was removed using a tonsil clamp and scissors. Hemostasis was achieved with gauze and gentle pressure for 5 minutes. If necessary for complete hemostasis electrosurgery was performed on bleeding vessels. Afterwards, patients were monitored in the postanesthetic care unit and discharged on the same day.

Postoperative Pain Medication

© 2025 Wong Chung JERE et al. *JAMA Network Open*.

Patients were provided postoperative pain relief with acetaminophen, 500 mg, as needed, up to 4 times daily (max 1000 mg per dose). If required, diclofenac, 50 mg, was also administered up to 3 times daily for the initial 3 days post-surgery. Tramadol was prescribed if acetaminophen and diclofenac did not provide adequate pain control.

Data collection

Data on tonsil-related symptoms, quality of life (measured with the 5-level EuroQol 5-Dimensions survey [EQ-5D] [5] including the visual analogue scale [EQ-VAS]), healthcare use, work productivity and activity impairment (measured with the Work Productivity and Activity Impairment [WPAI] questionnaire[6]), and overall satisfaction with the received treatment (assessed on a 0-100 mm Visual Analog Scale [VAS]) were collected one and two years after surgery through digital questionnaires. In addition, at two and six weeks, patients were asked when they felt fully recovered and when they returned to work. For the previously published short-term effectiveness analysis, patients who had not fully recovered, returned to work, or ceased analgesic medication within 14 days post-surgery were censored at that time point.[1] This approach ensured that short-term recovery comparisons were limited to the predefined 14-day window. Patients with recovery times longer than 14 days were included in the long-term follow-up analysis, and their economic impact was assessed based on six-week follow-up data to capture extended recovery experiences. . To handle missing data, multiple imputation was used to create 100 completed datasets, using logistic, ordered logistic and linear regression models with predictive mean matching.[7] Predictors were randomization, sex, age, EQ-5D utilities over time and the VAS for severity of throat complaints over time. Additionally, for repeated measures, that same measure at other timepoints was used as predictor. For some variables with limited variation, technical estimation problems were solved by reducing the number of predictors. Randomization was never excluded from the prediction model."

Economic evaluation

A cost-utility analysis was performed from a societal perspective, at price level 2023, with a two-year time horizon. Utility reflects the value of quality of life, on a scale anchored at 0 (=as bad as death) and 1 (=perfect health). Utility was calculated using the Dutch tariff for the EQ-5D[8] and the EQ-VAS with power transformation.[9] Quality-adjusted life years (QALYs) were calculated by the area under the utility curves over the follow-up period. The frequency of CO_2-laser TO and TE was assessed from the hospital administrations. A cost-price analysis was performed for both procedures. Other tonsil-related healthcare use was reported by patients and valued using Dutch reference prices, without discounting.[10] Absenteeism from work was calculated by the patient-reported time to return to work, up to six weeks after the procedure. Presenteeism at work was calculated by the time between self-reported return to work and return to normal self, multiplied by the degree of impediment to work according to the WPAI. Both absenteeism and presenteeism were valued at €286 per full day.[10] Cost-effectiveness acceptability curves were calculated as the one-sided p-value for the difference in net benefit, depending on the willingness to pay for a QALY ($NB = WTP \times QALY - Costs$). Three sensitivity analyses were performed, in which costs were limited to healthcare costs (instead of societal costs), patients without registered TE or CO_2-laser TO were assumed to have had TE (instead of assuming no procedure), and QALYs were calculated from the EQ-Visual Analog Scale (EQ-VAS, instead of the EQ-5D index score).

Statistical analysis

The target sample size was determined for previously published short-term outcomes of this study.[1] The calculation based on data from a prior non-randomized prospective study.[11] Using a 2-sided log-rank test with a total sample size of 190 patients (95 in each group), the study achieved 80.2% power at a .05 significance level. This allowed for the detection of a CO_2-laser TO median functional recovery time of 8 days, assuming the TE group median survival time was 13.5 days, within a total observation time of 14 days.

© 2025 Wong Chung JERE et al. *JAMA Network Open*.

Baseline demographic and clinical characteristics are presented as means with SDs, or as counts and percentages. Long-term clinical outcomes at one- and two-year after surgery were performed on an intention-to-treat basis (randomized patients analyzed according to randomization). Pooled means were compared months after surgery were compared using unpaired t-tests and proportions of binary outcomes were compared using logistic regression. Within the CO_2-laser TO and TE groups, changes from baseline were assessed using paired t-tests. Two-sided P values were computed, and a significance level of .05 was used for all testing. Statistical analyses were performed using SPSS, version 27 (IBM). External data monitoring was performed yearly to ensure data quality.

Patient Involvement

Members of the Patient Advisory Board of the Hagaziekenhuis hospital were actively involved in the development of the research questions, questionnaires, and recruitment strategy. They provided valuable feedback on the clarity and relevance of the study materials and consent forms. During data analysis, their perspectives helped interpret the results, ensuring that the findings aligned with patient experiences and priorities.

Role of the funding source

There was no external funding source for this study.

eReferences

1. Wong Chung JERE, van Geet R, van Helmond N, et al. Time to Functional Recovery After Laser Tonsillotomy Performed Under Local Anesthesia vs Conventional Tonsillectomy With General Anesthesia Among Adults: A Randomized Clinical Trial. *JAMA Network Open*. 2022;5(2):e2148655. doi:10.1001/jamanetworkopen.2021.48655

2. H.J. Rosingh. Richtlijn Ziekten van adenoïd en tonsillen (ZATT). Richtlijnendatabase.nl. 2014. https://richtlijnendatabase.nl/richtlijn/ziekten_van_adenoid_en_tonsillen_zatt/indicatie_voor_adenotomie_bij_zatt.html

3. Jong PTVM de. Gezondheidsraad: Commissie "Laserveiligheid in de gezondheidszorg." Published online 1992. Accessed August 9, 2020.

© 2025 Wong Chung JERE et al. *JAMA Network Open*.

https://pure.knaw.nl/portal/en/publications/gezondheidsraad-commissie-laserveiligheid-in-de-gezondheidszorg

4. Wong Chung J, van Helmond N, van Geet R, van Benthem P, Blom H. CO2-Lasertonsillotomy Under Local Anesthesia in Adults. *J Vis Exp*. 2019;(153). doi:10.3791/59702

5. Herdman M, Gudex C, Lloyd A, et al. Development and preliminary testing of the new five-level version of EQ-5D (EQ-5D-5L). *Qual Life Res*. 2011;20(10):1727-1736. doi:10.1007/s11136-011-9903-x

6. Reilly MC, Zbrozek AS, Dukes EM. The Validity and Reproducibility of a Work Productivity and Activity Impairment Instrument. *PharmacoEconomics*. 1993;4(5):353-365. doi:10.2165/00019053-199304050-00006

7. Buuren S van. *Flexible Imputation of Missing Data, Second Edition*. 2nd ed. Chapman and Hall/CRC; 2018. doi:10.1201/9780429492259

8. M Versteegh M, M Vermeulen K, M A A Evers S, de Wit GA, Prenger R, A Stolk E. Dutch Tariff for the Five-Level Version of EQ-5D. *Value Health*. 2016;19(4):343-352. doi:10.1016/j.jval.2016.01.003

9. Stiggelbout AM, Eijkemans MJ, Kiebert GM, Kievit J, Leer JW, De Haes HJ. The “utility” of the visual analog scale in medical decision making and technology assessment. Is it an alternative to the time trade-off? *Int J Technol Assess Health Care*. 1996;12(2):291-298. doi:10.1017/s0266462300009648

10. Hakkaart-van Roijen L, van der Linden N, Bouwmans CAM, Kanters T, Tan SS. Kostenhandleiding, Methodologie van kostenonderzoek en referentieprijzen voor economische evaluaties in de gezondheidszorg. English: Costing manual: Methodology of costing research and reference prices for economic evaluations in healthcare. Published online 2015. Accessed May 23, 2024. https://docplayer.nl/12082781-Kostenhandleiding-methodologie-van-kostenonderzoek-en-referentieprijzen-voor-economische-evaluaties-in-de-gezondheidszorg.html

11. Lourijsen ES, Wong Chung JERE, Koopman JP, Blom HM. Post-operative morbidity and 1-year outcomes in CO2-laser tonsillotomy versus dissection tonsillectomy. *Acta Oto-Laryngologica*. 2016;136(10):983-990. doi:10.1080/00016489.2016.1183040

© 2025 Wong Chung JERE et al. *JAMA Network Open.*

eTable 1. Baseline demographic and clinical characteristics in tonsillotomy and tonsillectomy groups.

	Tonsillotomy (n = 98)	Tonsillectomy (n = 98)
Demographic characteristics		
Sex: M/F n (%)		
Male	29 (30)	31 (32)
Female	69 (70)	67 (68)
Age in years, mean ± SD	29 ± 10	30 ± 8
Smoking status, n (%)		
Current	17 (18)	14 (14)
Former	24 (25)	16 (16)
Never smoked	56 (58)	46 (47)
Tonsil symptoms		
Chief tonsil complaint, n (%)		
Sore throat without fever	31 (32)	31 (32)
Sore throat with fever	33 (34)	33 (34)
Tonsillolithiasis	32 (33)	31 (32)
Snoring	2 (2)	2 (2)
Dysphagia	0 (0)	1 (1)
Self-reported severity of tonsil complaints (ordinal), n (%)		
Minimal	1 (1)	1 (1)
Mild	21 (22)	18 (18)
Moderate	59 (61)	47 (48)
Severe	16 (16)	10 (10)

© 2025 Wong Chung JERE et al. *JAMA Network Open*.

Self-reported severity of tonsil complaints (continuous) in mm, mean ± SD	57 ± 19	59 ± 17
Quality of life and work/activity impairment		
QoL (EQ-5D-5L) index score, median (IQR)	0.87 (0.81 – 1.00)	0.87 (0.84 – 1.00)
EQ-5D-5L general health rating, median (IQR)	80 (70 – 89)	80 (70 – 89)
Employed, n (%)	70 (74)	57 (58)
WPAI overall work impairment in %, median (IQR) [a]	7 (2 – 12)	5 (0 – 11)
WPAI interference with daily activities (0-10), median (IQR)	3 (2 – 6)	4 (2 – 6)

EQ-5D-5L = Euroqol 5 dimensions quality of life (QoL) survey

WPAI = Work Productivity and Activity Impairment Questionnaire

a: WPAI is only evaluated in patients who are employed

© 2025 Wong Chung JERE et al. *JAMA Network Open*.

eTable 2: Cost price of the tonsillectomy and CO_2-laser tonsillotomy procedures (in €)

	Tonsillectomy	Laser tonsillotomy
**Pre-procedure 10-minute outpatient visit[1]**	197	197
**Pre-operative anesthetic assessment**	89	
**Operating room[2] - 60 minutes**	841	
**Day-care admission - bed occupancy 120 minutes**	585	
**Outpatient personnel[3] – 45 minutes**		136
**Alterations to the outpatient treatment room[4]**		15
**Laser equipment[5]**		123
**Laser maintenance[6]**		29
**Laser materials**		59
**Post-procedure 10-minute outpatient visit**	112	112
**Total costs per procedure**	1824	671

1. A pre-procedure outpatient visit was also counted for patients who did not undergo either procedure, but not for repeat CO_2-laser tonsillotomy
2. Including personnel
3. Physician plus an assistant
4. Assuming 25,000 euro, distributed over 2000 patients during 20 year
5. Assuming 105,000 euro, distributed over 1000 patients during 10 year
6. Assuming 2,500 euro annually, distributed over 100 patients

© 2025 Wong Chung JERE et al. *JAMA Network Open*.

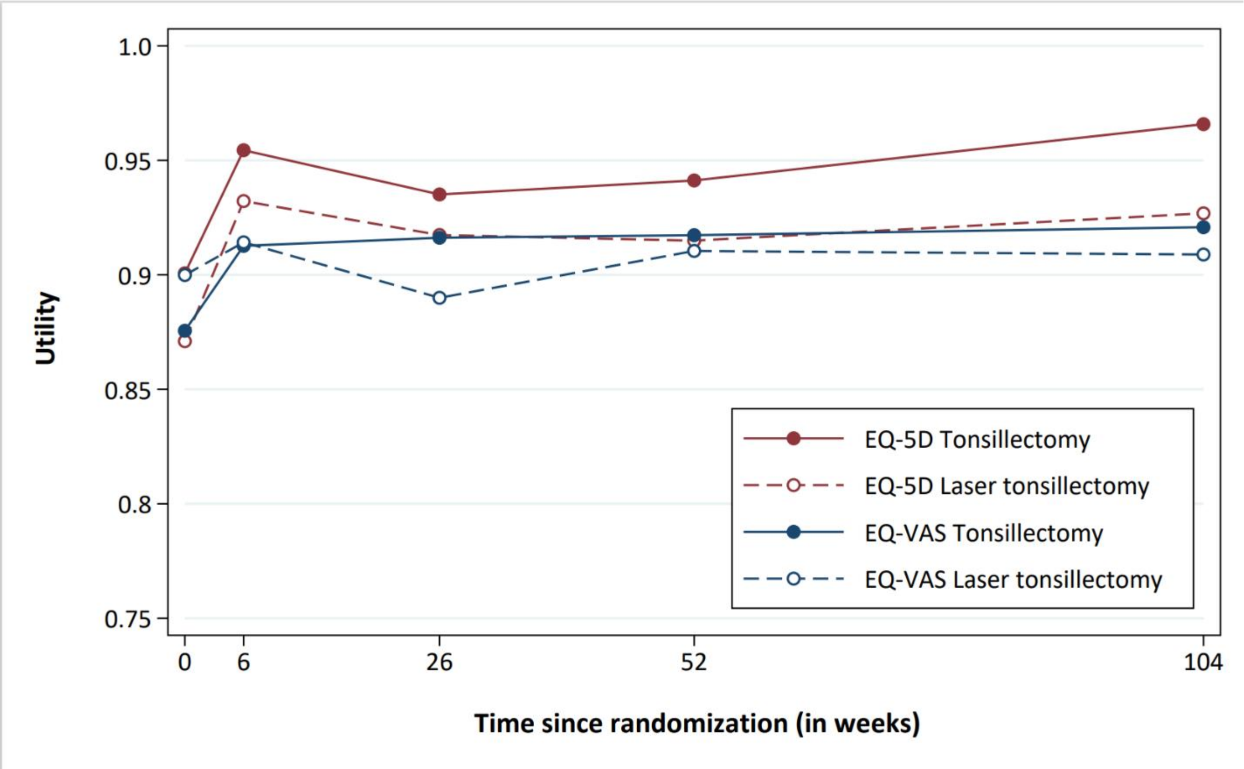

eFigure. Utilities over time, by randomization group.

© 2025 Wong Chung JERE et al. *JAMA Network Open*.
