# Supplementary material for: Long-Term Efficacy and Cost-Effectiveness of Laser Tonsillotomy vs Tonsillectomy: A Secondary Analysis of a Randomized Clinical Trial
Source: JAMA Netw Open. 2025 Apr 29;8(4):e254858. doi: 10.1001/jamanetworkopen.2025.4858 (PMC12042055; doi:10.1001/jamanetworkopen.2025.4858)
Supplement: Supplement 3. — Data Sharing Statement [file jamanetwopen-e254858-s003.pdf]

Data Sharing Statement

Wong Chung. One- and 2-Year Outcomes and Cost-Effectiveness of Laser Tonsillotomy With Local Anesthesia vs Tonsillectomy With General Anesthesia. *JAMA Netw Open*. Published April 16, 2025. doi:10.1001/jamanetworkopen.2025.4858

Data

Additional Information: NetherlandsTrialRegister, NTR7044, https://onderzoekmetmensen.nl/nl/trial/35754
Data available: Yes
Data types: Deidentified participant data
How to access data: All data collected in this study will be made available in a timely fashion to the scientific community for use in joint analyses on reasonable request after approval of the local Research Ethics Committee and with a signed data access agreement. All patient data is stored in an electronic data capture system. Contact info: h.blom@hagaziekenhuis.nl
When available: With publication

Supporting Documents

Document types: None

Additional Information

Who can access the data: All data collected in this study will be made available in a timely fashion to the scientific community for use in joint analyses on reasonable request after approval of the local Research Ethics Committee and with a signed data access agreement. All patient data is stored in an electronic data capture system. Contact info: h.blom@hagaziekenhuis.nl
Types of analyses: For additional analysis meta analysis
Mechanisms of data availability: Access to EC system after approval
Any additional restrictions: -
